# Modulating alfalfa growth: impacts of plant growth regulators on physiology, architecture, and seed yield

**DOI:** 10.1186/s12870-026-08173-x

**Published:** 2026-02-06

**Authors:** Xinyao Wang, Yixin Liu, Xiaoqing Sui, Kaihui Li, Amanula Yimingniyazi, Lianwu Jin, Mengqing Lang

**Affiliations:** 1 College of Grassland Science, Xinjiang Agricultural University, Ürümqi, China; 2 Key Laboratory of Grassland Resources and Ecology of Western Arid Region, Ministry of Education, Ürümqi, China; 3 Xinjiang Key Laboratory of Grassland Resources and Ecology, Urumqi, Xinjiang 830052 China; 4 Xinjiang Institute of Ecology and Geography, Chinese Academy of Sciences, Urumqi, 830011 Xinjiang China

**Keywords:** Plant growth regulator, Cell Ultrastructure, Photosynthetic Capacity, Carbon and nitrogen metabolism balance, Plant Shape

## Abstract

**Background:**

Alfalfa seed yield is severely restricted by its indeterminate growth habit, with actual average yield only reaching 4% of potential yield in field production. Plant growth regulators (PGRs) can modulate the balance between vegetative and reproductive growth, yet research on PGR application for alfalfa plant architecture optimization and seed yield improvement remains limited. To address this gap, this study treated “XinMu No.4” alfalfa (*Medicago sativa* L.) with three PGRs at specific concentration gradients: flumetralin (CAG: 0.5/1.0/1.5 g L^− 1^), 1,1-dimethyl-piperidinium chloride (DPC: 0.25/0.35/0.45 g L^− 1^), and compound sodium nitrophenolate (CSN: 0.15/0.20/0.25 g L^− 1^), to investigate their effects on growth, photosynthetic characteristics, carbon-nitrogen metabolism, and seed yield.

**Results:**

Key findings showed that a single spray of 0.35 g L^− 1^DPC resulted in complete cell structure, significantly increased chloroplast volume (by 23.6%), enhanced starch granule number (by 41.2%), and improved photosynthetic capacity (chlorophyll content increased by 47.5% and net photosynthetic rate (Pn) increased by 32.8%). This treatment also elevated nitrate reductase activity by 37.5% and sucrose synthase activity by 29.4%, enhancing carbon-nitrogen metabolic capacities. By increasing branch number to 63 branches/plant, inflorescences per plant increased by approximately 28 Pcs/Plant, ultimately boosting seed yield to 329.76 kg·hm^− 2^. Comprehensive membership function evaluation indicated this treatment had the highest average membership degree (0.918).

**Conclusion:**

Comprehensive analysis showed that 0.35 g L^− 1^ DPC was the most effective in shaping plant architecture and boosting seed yield.

**Supplementary Information:**

The online version contains supplementary material available at 10.1186/s12870-026-08173-x.

## Introduction

Alfalfa (*Medicago sativa* L.), known as the “king of forages“ [1], is associated with its indeterminate growth(referring to the maintenance of apical meristem activity) habit; however, excessive nutrient application often results in low seed yield as only a small fraction of flowers develop into mature pods. In field production, uncontrolled vegetative growth restricts reproductive allocation: the actual average seed yield is merely 4% of its potential yield, with the highest ≤ 18% [2].

Balancing vegetative and reproductive growth is critical for optimizing land resource utilization, crop quality, and yield [3, 4]. Plant growth regulators (PGRs) modulate physiological metabolism, morphological structure, and nutrient allocation to regulate growth and development [5]. For instance, brassinosteroids enhance crop resilience (cold, disease, salt resistance) and yield(These have been validated in *Arabidopsis thaliana*) [6]. PGRs also boost photosynthetic capacity and nutrient absorption, improving crop quality [7, 8]. In soybean (*Glycine max*), PGRs influence leaf physiology via enzyme pathways, affecting yield [9]. Field studies confirm these effects: four PGRs applied to cotton increased growth and photosynthetic parameters [10]; peanut pod injection improved leaf chlorophyll and yield [11]; Jun jujube leaf spraying enhanced photosynthesis and fruit quality [12]. Thus, PGRs are widely used to optimize plant growth and production.

The plant growth regulators (PGRs) flumetralin (CAG, efficient growth retardant), 1,1-dimethyl-piperidinium chloride (DPC, plant growth retardant), and compound sodium nitrophenolate (CSN, powerful cell agent) have been applied to regulate plant growth. CAG is quickly absorbed, increases the number of compact branches, greatly improves field ventilation and light conditions, improves whole canopy light conditions, decreases the premature failure rate, extends the photosynthesis time of lower leaves, promotes precocious single boll weight, prevents empty fruit branches, and improves seed yield. Dai et al. [13] found that spraying CAG at different concentrations had a strong effect on the agronomic characteristics and yield of sea island cotton (*Gossypium barbadense*). Wang et al. [14] showed that spraying CAG improved cotton plant strains and increased cotton yield. DPC is absorbed by the plant leaves and roots and then transferred to the whole plant, and it has been shown to adjust the growth of crops by preventing plant growth and improving seed yield. Li et al. [15] showed that spraying DPC on cotton improved cotton plant strains by increasing cotton yield. Xu et al. [16] also found that DPC effectively coordinated the balance between vegetative growth and reproductive growth, with reasonable spraying helping to promote the accumulation of dry matter in the reproductive organs of cotton plants. CSN is a powerful cell agent with a chemical composition of 5-nitro guaiol sodium, sodium o-nitrophenol, and sodium p-nitrophenol. After contact, CSN can quickly penetrate the plant, and then the photosynthetic products are quickly transported to the fruit, where they regulate the growth of crops and improve plant growth and yield. An Xia et al. [17] found that injecting 6 mg L^-1^ sodium nitrosol solution increased the accumulation of dry matter in two varieties of wheat and significantly increased production. Yang et al. [18] showed that after spraying N, CSN, and a combination of CSN and N during the flowering period of alfalfa, the soluble sugar, starch, and soluble protein contents and antioxidant enzyme activity in the leaves increased, and seed yield improved.

Both experimental and production practices have demonstrated that yield and potential yield can be significantly improved by enhancing apical dominance and regulating the relationship between vegetative and reproductive growth. The bud stage is a critical period for plant type formation. Therefore, plant type shaping and artificial regulation during the branching stage of legumes such as alfalfa are expected to achieve good results, and such research has important theoretical significance and practical application value. Currently, there is a lack of systematic research on the physiological mechanisms underlying low seed yield in alfalfa and the concentration effects of PGR regulation, with most existing studies focusing on single regulators and having inconsistent concentration gradient settings [13–18]. Plant growth regulators can balance vegetative and reproductive growth, promote the normal growth and development of plant organs, thereby increasing seed yield. However, the synergistic regulatory mechanisms of different concentrations of PGRs (CAG 0.1%~0.5%, DPC 20–100 mg L⁻¹, CSN 2–10 mg L⁻¹) on alfalfa plant type shaping and yield formation remain unclear. Therefore, this study used alfalfa as experimental material, after preliminary experiments, and adopted three concentration gradient treatments of CAG (0.5 g L^− 1^, 1.0 g L^− 1^, 1.5 g L^− 1^), DPC (0.25 g L⁻¹, 0.35 g L⁻¹, 0.45 g L^− 1^), and CSN (0.15 g L⁻¹, 0.20 g L^− 1^, 0.25 g L^− 1^) to reveal the mechanisms regulating enzyme activity and seed yield. The results provide a reference basis for the rational application of plant growth regulators in alfalfa seed production with concentration parameters.

## Material methods

### Plant materials

In this experiment, “XinMu No. 4” alfalfa (*Medicago sativa* L.) was used as the experimental material. The seeds were purchased from the College of Grass Industry of Xinjiang Agricultural University, Xinjiang, China.

### Experimental site conditions and reagent concentrations

The experimental plots were arranged in randomized blocks, and the plot area was set to 4 m × 2 m according to local practice. The sowing rows were spaced 60 cm apart, the plants were spaced 20 cm apart, and three to four seeds were sown per hole. The trial was conducted in May 2023 (second year after sowing), and the treatment concentrations are shown in Supplementary materials(Table S1). Throughout the reproductive period, timely weeding and pest and disease control were conducted. The three experimental treatments included one foliar spraying of each PGR (CAG, DPC, and CSN). The control treatment (CK) consisted of primary foliar spraying of water. Three replications of the treatment plots were performed. Treatments were applied at the branching stage, and the amount of spray solution was 250 ml·m^-2^. Six plots were established for each concentration, and a total of 60 plots were sprayed. The second treatment was conducted 7 d after the first application based on the results of the first spraying (54 plots were treated in the first spraying, and 27 plots were treated in the second spraying). The treatment concentration remained unchanged, and three plots were sprayed for each concentration, with the steps consistent with that of the first application. Each plot was sprayed with PGRs after 19:30 pm on a clear, windless, partly cloudy day, and the treatments were manually applied using a hand-held spray can.

### Ultrastructural observation of leaf cells

At 7 d after PGR treatment, 1 mm × 3 mm samples of leaf pulp tissue from the middle leaf blade were collected from the apical to basal 2nd to 4th leaves. Three alfalfa plants were randomly collected from each plot, and the samples were fixed in 5% glutaraldehyde solution immediately after sampling and stored at 4 ℃. The samples stored at 4 ℃ were washed three times with phosphate buffer (pH: 7.6) for 20 min each. The material was then transferred to 1% osmium tetroxide and fixed in distilled water for 4 h at 4 ℃. Next, the material was transferred to 1% osmium acid, fixed at 4 ℃ for 4 h, and washed with distilled water three times for 20 min each. Subsequently, the samples were dehydrated by gradient ethanol, embedded in epoxy resin 618, sectioned using a LKB-V ultrathin microtome (LKB-V, LKB, Norway, Sweden), double-stained with diuronic acetate and lead citrate, and observed and imaged under a Hitachi H-7600 transmission electron microscope (Sinaida Precision Instrument [Shanghai] Co., LTD) [19].

### Determination of photosynthesis indexes

The middle leaf blade of the apical to basal 2nd to 4th leaves was placed into the leaf chamber of a CIRAS-3 photosynthesizer (PP Systems, Inc, Amesbury, MA 01913, U.S.A.), and the leaf was clamped until the data were stabilized (1–3 min) to record the net photosynthetic rate (Pn), leaf intercellular CO_2_ concentration (Ci), leaf transpiration rate (Tr), and leaf stomatal conductance (Gs). The chlorophyll content was determined using a SPAD-502 chlorophyll meter.

### Determination of carbon and nitrogen metabolism enzymes and metabolites

Sampling was conducted 7 d after the first(May 6, 2024) and second(May 13, 2024) spraying of PGRs. Thirty grams of middle leaves (apical to basal 2nd-4th) were collected, wrapped in tinfoil, labeled, quick-frozen in liquid nitrogen for 20 min, and stored at − 80 ℃. For enzyme activity analysis, active enzymes were measured using kits from Nanjing Aoqing Bio-technology Co. Absorbance was determined with an enzyme labeler after color development to calculate activity. For metabolite analysis, samples were dried, sieved through a 60-mesh sieve, and assessed for routine chemical composition (sucrose, starch, soluble protein) using kits from the same company, with absorbance measured via enzyme markers.

### Measurement of agronomic traits

Absolute plant height (cm) was measured for 30 alfalfa plants selected at the branching, bud, and full bloom stages.

The number of branches per plant was counted for 30 alfalfa plants per plot.

Measurements were performed on 30 randomly selected plants at the seed maturity stage within the sampling rows, and all inflorescences and the corresponding number of pods of the plants were counted separately.

The number of inflorescences/plant was counted for the 30 alfalfa plants.

When 80% of the pods had blackened in each plot, they were bagged after flush mowing. The pods were then dried, manually threshed, and cleaned. Weighing was performed using a 1/100 balance to calculate the seed yield in kg·hm^-2^.

### Data analysis

Raw data were organized using Excel 2021 software, and SPSS 26.0 software was used for datamultiple comparisons usingone-way analysis of variance (one-way-anova), and Duncan’s test was applied for variance analysis. All treatments included at least three independent biological replicates. Pvalue < 0.05was considered statistically significant. Graphs were plotted using Origin 2024 software, and structural equation modeling (SEM) for path analysis was performed using the piecewiseSEM package in SmartPSL software.

## Results

### Effect of plant growth regulator treatments on ultrastructure of alfalfa leaf cells

#### Effect of Flumetralin on ultrastructure of alfalfa leaves

**Fig. 1:**
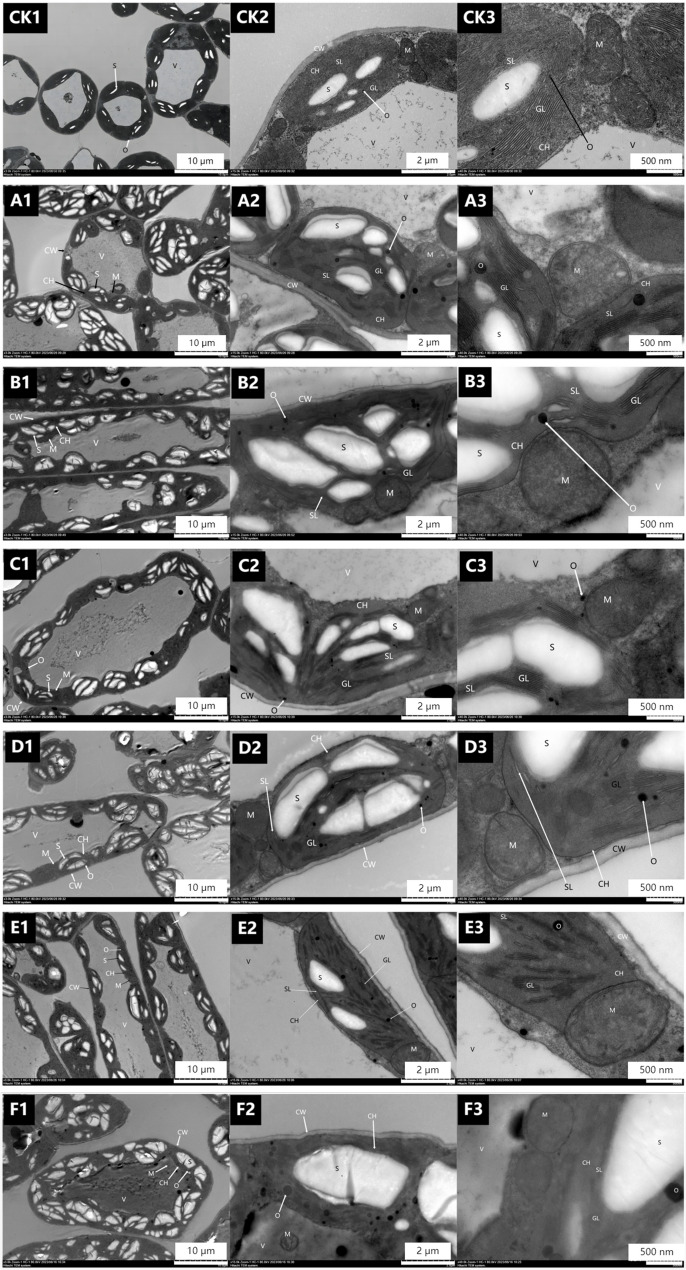
Effects of spraying different concentrations of CAG on the cellular ultrastructure of alfalfa leaves. Note: This figure shows Electron Microscopy (EM) images of the same leaf at different magnifications.For one spray treatment: CK (water spray control), **A** 0.5 g L− 1 CAG, (**B**) 1.0 g L− 1 CAG, (**C**) 1.5 g L− 1 CAG; For two spray treatments: (**D**) 0.5 g L− 1 CAG, (**E**) 1.0 g L− 1 CAG, (**F**) 1.5 g L− 1 CAG; In the figure, the number 1 in the top-left corner represents 10 μm; 2 represents 2 μm; 3 represents 500 nm. CW: cell wall; CH: chloroplast; Cylakoids: GL (matrix lamellar), SL (matrix lamellar); S: starch grain; O: osmiophilic granules; M: mitochondria; V: vacuole.

Figure 1 shows the alfalfa mesophyll cell ultrastructure after one spray of CAG. At 1.0 g L^-1^ CAG (Fig. 1B1, B2, and B3), the number of thylakoids was greater than that of the CK and 0.5 g L^-1^ CAG treatments (Fig. 1B2). Moreover, the matrix was stacked (Fig. 1B3), the volume of chloroplasts increased, the number of starch granules increased (Fig. 1B1 and B2), the number of osmiophilic granules increased, and the volume was slightly larger than that of the CK (Fig. 1B2 and B3). The mitochondrial bilayer membrane structure and cristae structure were clear (Fig. 1B3), although the cells began to show irregular bodies (Fig. 1B1). The CAG_2_ treatment was generally better than the CAG_1_ treatment, the initial CAG_3_ treatment, and the second spray of CAG_1_, CAG_2_, and CAG_3_. (See “Materials and Methods” for information on the different processing methods).

#### Effect of 1,1-DPC on ultrastructure of alfalfa leaves

**Fig. 2:**
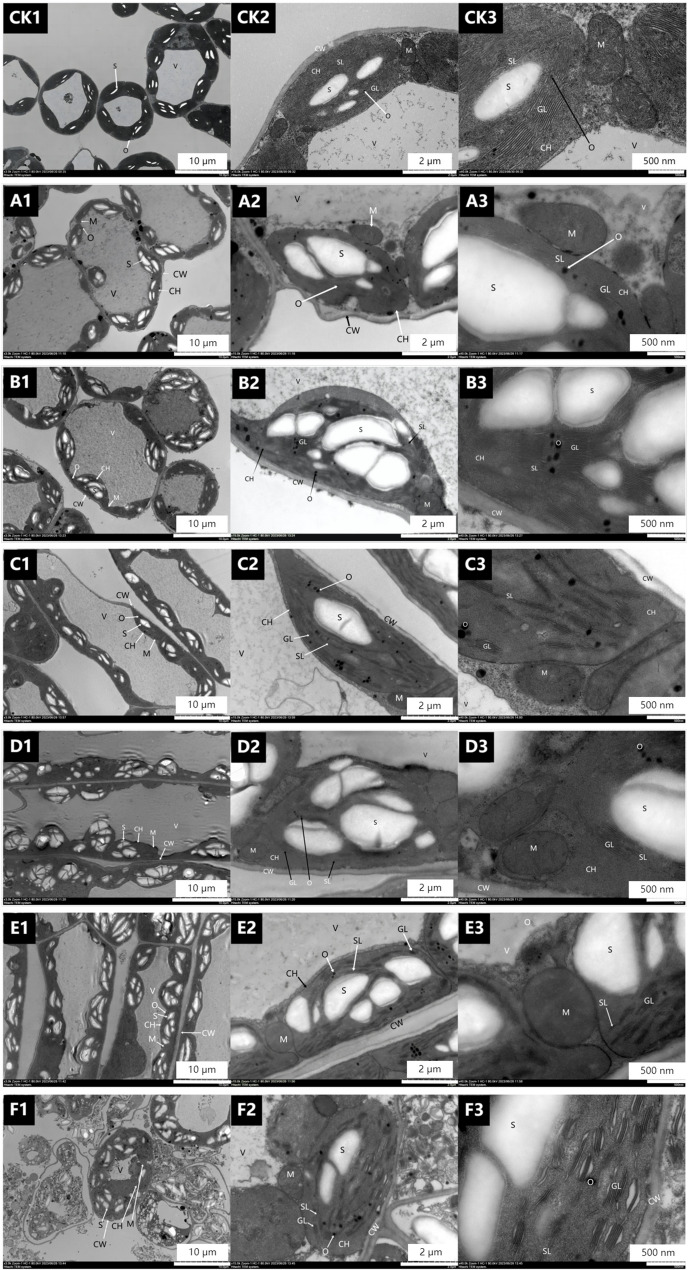
Effects of spraying different concentrations of DPC on the cellular ultrastructure of alfalfa leaves. Note: This figure shows Electron Microscopy (EM) images of the same leaf at different magnifications.For one spray treatment: CK (water spray control), **A** 0.25 g L^− 1^ DPC, (**B**) 0.35 g L^− 1^ DPC, (**C**) 0.45 g L^− 1^ DPC; For two spray treatments: (**D**) 0.25 g L^− 1^ DPC, (**E**) 0.35 g L^− 1^ DPC, (F) 0.45 g L^− 1^ DPC; See Fig. 1 for other content

The ultrastructure of alfalfa mesophyll cells after one spray of DPC is shown in Fig. 2. For 0.35 g L^-1^ DPC, the number of grana thylakoids was greater than that of the CK (Fig. 2B2). In addition, matrix lamina stacking was increased, the chloroplast volume was increased, and the number of starch grains was higher relative to that of the CK (Fig. 2B1, B2, and B3). The number of osmophilic particles also increased significantly compared with that of the other treatments, and the volume was larger than that of the CK (Fig. 2B2), the mitochondrial bilayer membrane structure and cristae structure were clear (Fig. 2B3), and the cells were full (Fig. 2B1). These results were overall superior to those of DPC_1_ and DPC_3_ and a second spray of DPC_1_, DPC_2_, and DPC_3_.

#### Effect of spraying CSN on ultrastructure of alfalfa leaf cells

**Fig. 3:**
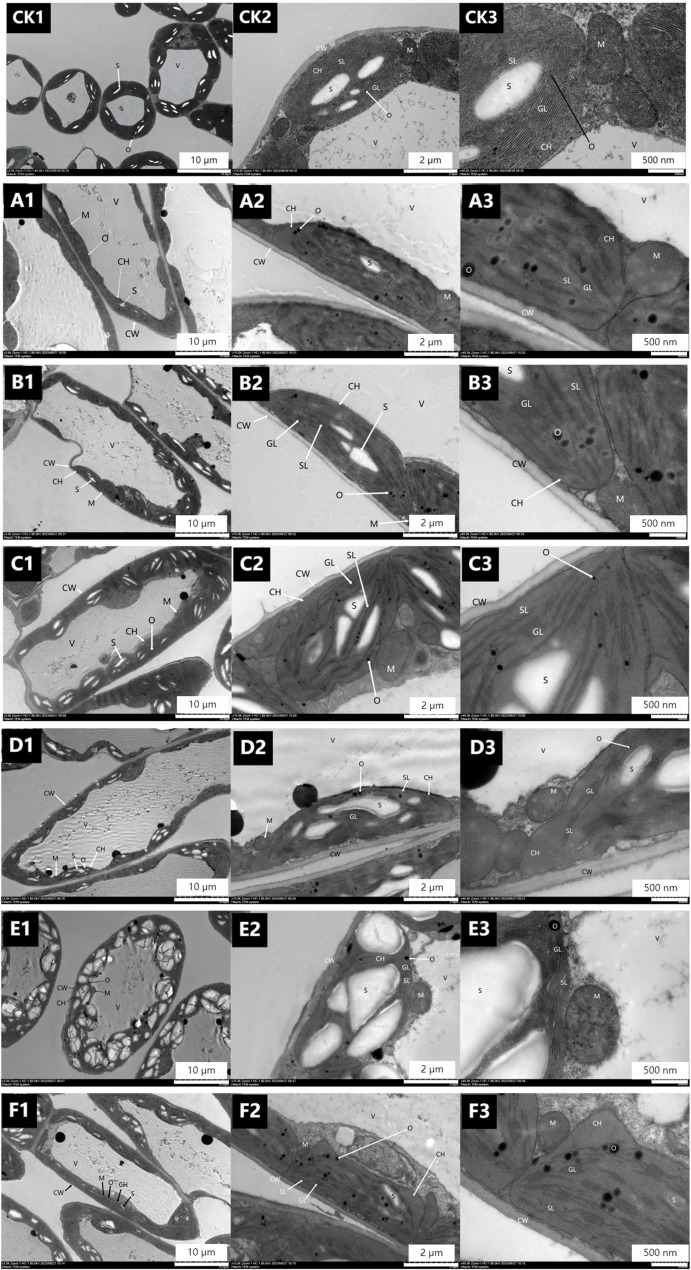
Effects of spraying different concentrations of CSN on the cellular ultrastructure of alfalfa leaves. Note: This figure shows Electron Microscopy (EM) images of the same leaf at different magnifications.For one spray treatment: CK (water spray control), **A** 0.15 g L^− 1^ CSN, (**B**) 0.2 g L^− 1^ CSN, (**C**) 0.25 g L^− 1^ CSN; For two spray treatments: (**D**) 0.15 g L^− 1^ CSN, (**E**) 0.2 g L^− 1^ CSN, (**F**) 0.25 g L^− 1^ CSN; See Fig. 1 for other content

The ultrastructure of alfalfa mesophyll cells after CSN application is shown in Fig. 3. When the plants were sprayed a second time with 0.25 g L^− 1^ CSN (Fig. 3B1, B2, and B3), the basal lamellae were stacked, chloroplast volume increased, and the number of starch grains increased (Fig. 3B2 and B3); furthermore, the number of osmophilic particles increased significantly compared with that of the CK, the volume was slightly larger than that of the CK (Fig. 3B2 and B3), and the mitochondrial bilayer membrane structure and cristae structure were clear (Fig. 3B2). Overall, this treatment was superior to one spray of the three treatments and the second spray of CSN_1_ and CSN_2_.

### Effect of plant growth regulators treatments on photosynthetic capacity of alfalfa

**Fig. 4:**
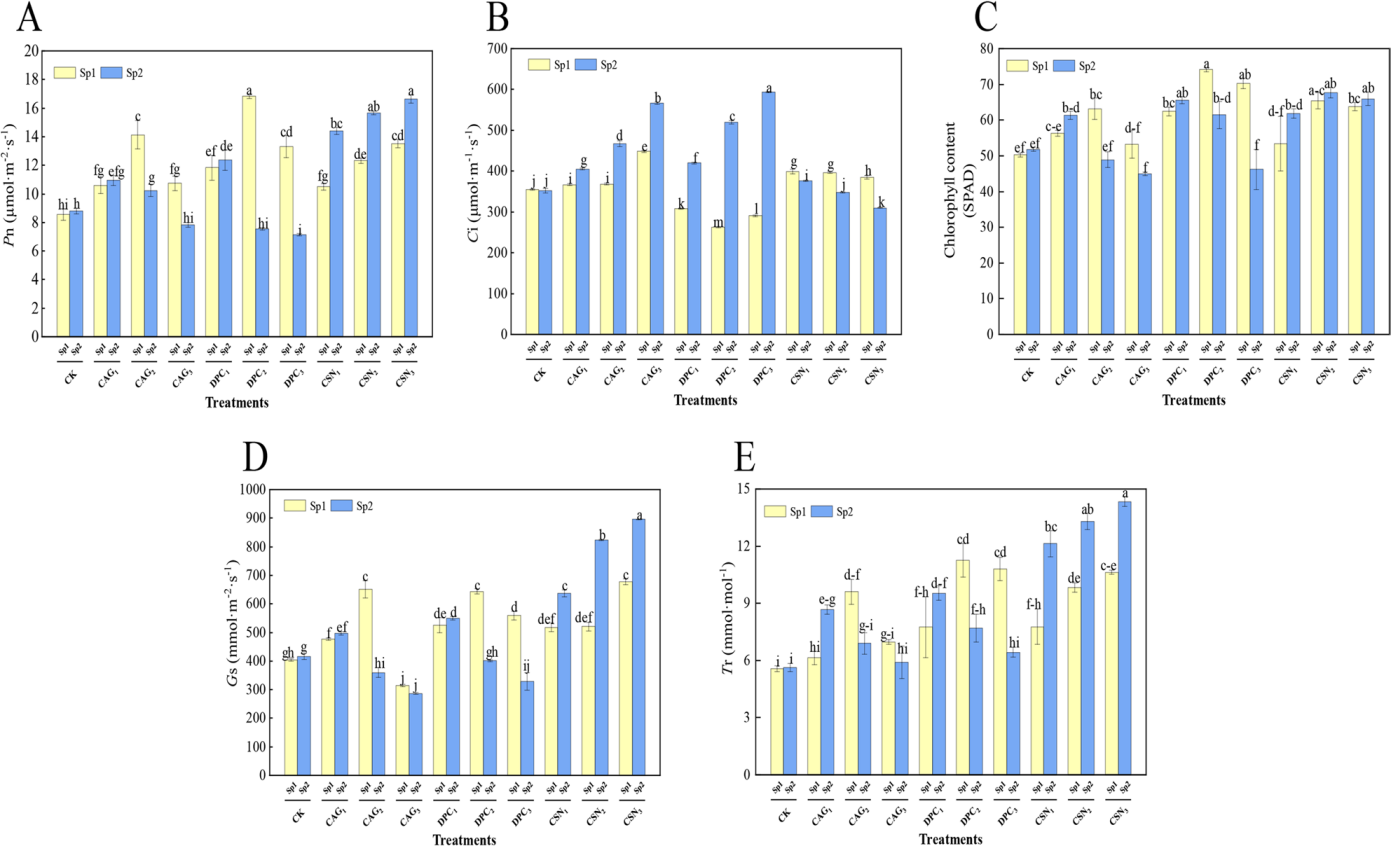
Effect of spraying PGRs on intercellular CO_2_ concentration in alfalfa leaves. Note: Sp1: primary spray; Sp2: secondary spray; (CK) Water spray; (CAG_1_) 0.5 g L^− 1^ CAG; (CAG_2_) 1.0 g L^− 1^ CAG; (CAG_3_) 1.5 g L^− 1^ CAG; (DPC_1_) 0.25 g L^− 1^ DPC; (DPC_2_) 0.35 g L^− 1^ DPC; (DPC_3_) 0.45 g L^− 1^ DPC; (CSN_1_) 0.15 g L^− 1^ CSN, (CSN_2_) 0.2 g L^− 1^ CSN, (CSN_3_) 0.25 g L^− 1^ CSN. Error bars represent standard deviation (SD); One-way ANOVA was used; Different lowercase letters indicate significant differences between groups (*P* < 0.05)

As shown in Fig. 4A, the Pn of the leaves treated with DPC_2_ showed a trend of initially increasing and then decreasing (by up to 16.83 µmol·m^− 2^·s^− 1^), and the value was significantly higher than the Pn of the CK (*P* < 0.05). The concentration of intercellular CO_2_ after one spray of the DPC treatment showed an initially decreasing and then increasing trend (Fig. 4B). The lowest intercellular CO_2_ concentration of the DPC_2_ treatment was 262.67 µmol·m^− 1^·s^− 1^ which was significantly less than that of the CK (*P* < 0.05). Moreover, the chlorophyll content of the DPC treatment tended to increase first and then decrease, and after the second spray of DPC, it decreased continuously (Fig. 4C). Specifically, the chlorophyll content of the first spray of the DPC_2_ treatment reached the highest value of 74.13 SPAD, which was strongly significantly different from that of the CK (*P* < 0.05). The stomatal conductance after a second spray of the CSN treatment showed a continuous increase, with the stomatal conductance reaching 895.67 mmol·m^− 2^·s^− 1^ after the CSN_3_ treatment, which was significantly different from that of the CK (*P* < 0.05) (Fig. 4D). The Tr after a second CSN treatment showed a continuous increase by up to 9.6 mmol·mol^− 1^ after the CSN_3_ treatment, which was significantly different from that of the CK (*P* < 0.05) (Fig. 4E). However, it is important to note that these positive effects were observed in short-term treatments. Long-term PGR applications may pose potential side effects on photosynthetic efficiency such as reduced chlorophyll stability or altered stomatal regulation over time which could undermine sustained performance. Additionally, further research is needed to explore whether prolonged use exhibits a diminishing returns effect, where incremental benefits to photosynthetic capacity decrease with continuous application despite initial positive outcomes.

### Effect of plant growth regulators treatments on activity of carbon metabolism enzymes in alfalfa

**Fig. 5:**
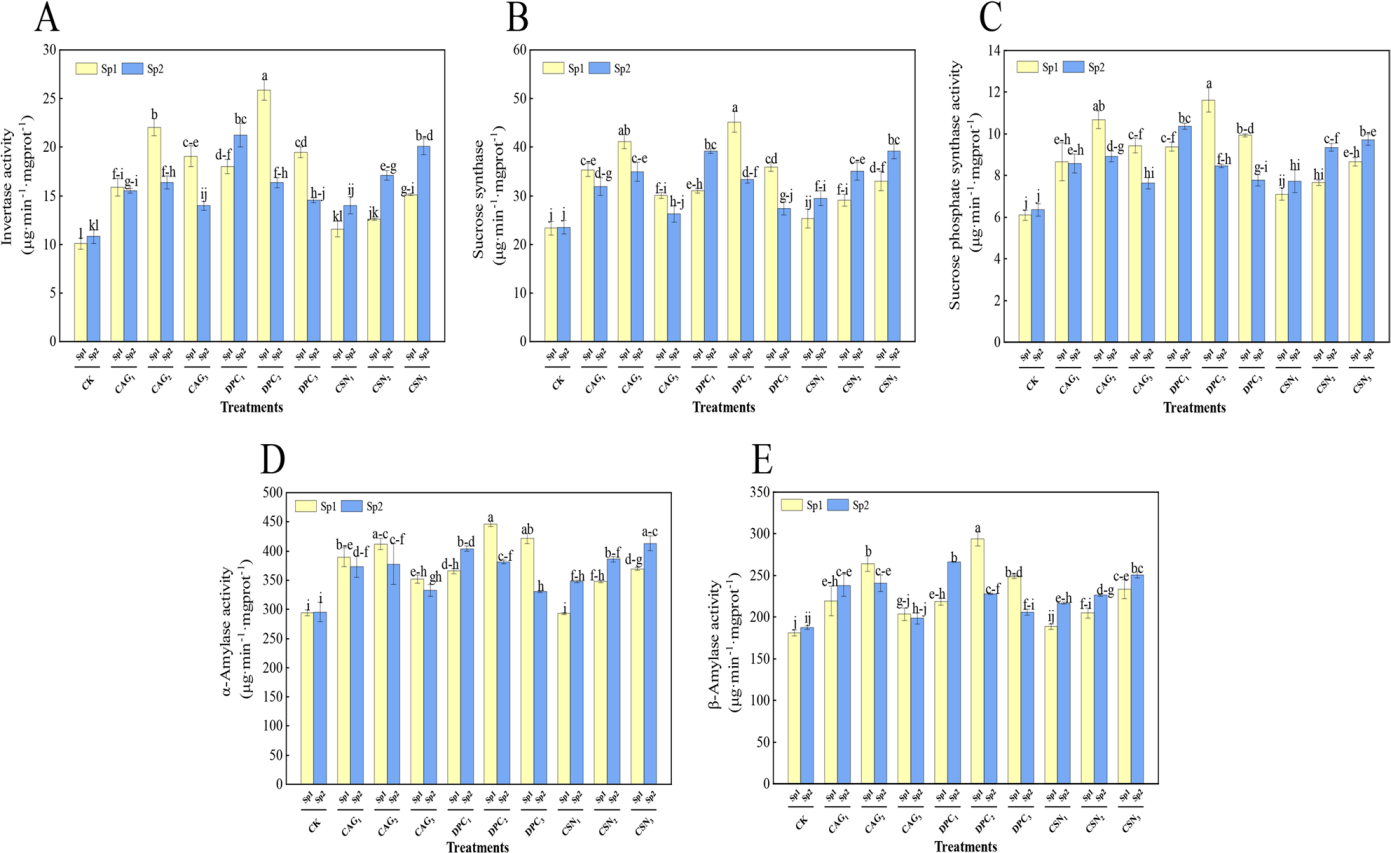
Effect of spraying PGRs on the activity of carbon metabolism enzymes in alfalfa. Note: Sp1: primary spray; Sp2: secondary spray; (CK) Water spray; (CAG_1_) 0.5 g L^− 1^ CAG; (CAG_2_) 1.0 g L^− 1^ CAG; (CAG_3_) 1.5 g L^− 1^ CAG; (DPC_1_) 0.25 g L^− 1^ DPC; (DPC_2_) 0.35 g L^− 1^ DPC; (DPC_3_) 0.45 g L^− 1^ DPC; (CSN_1_) 0.15 g L^− 1^ CSN, (CSN_2_) 0.2 g L^− 1^ CSN, (CSN_3_) 0.25 g L^− 1^ CSN. Error bars represent standard deviation (SD); One-way ANOVA was used; Different lowercase letters indicate significant differences between groups (*P* < 0.05)

As shown in Fig. 5, the sucrose invertase, sucrose synthetase, sucrose phosphate synthase, α-amylase, and β-amylase activity increased and then decreased successively after one spray of DPC, with the values significantly different (*P* < 0.05) from that of the CK. For the DPC_2_ treatment, sucrose invertase activity reached 25.88 µg·min^-1^·mgprot^-1^ (Fig. 5A); sucrose synthase activity reached 45.18 µg·min^-1^·mgprot^-1^ (Fig. 5B); sucrose phosphate synthetase activity reached 11.62 µg·min^-1^·mgprot^-1^(Fig. 5C), which was the highest; α-amylase activity reached 446.36 µg·min^-1^·mgprot^-1^ (Fig. 5D); and β-amylase activity reached 293.89 µg·min^-1^·mgprot^-1^ (Fig. 5E).

### Effect of plant growth regulators treatments on activity of nitrogen metabolism enzymes and carbon-nitrogen metabolites in alfalfa

**Fig. 6:**
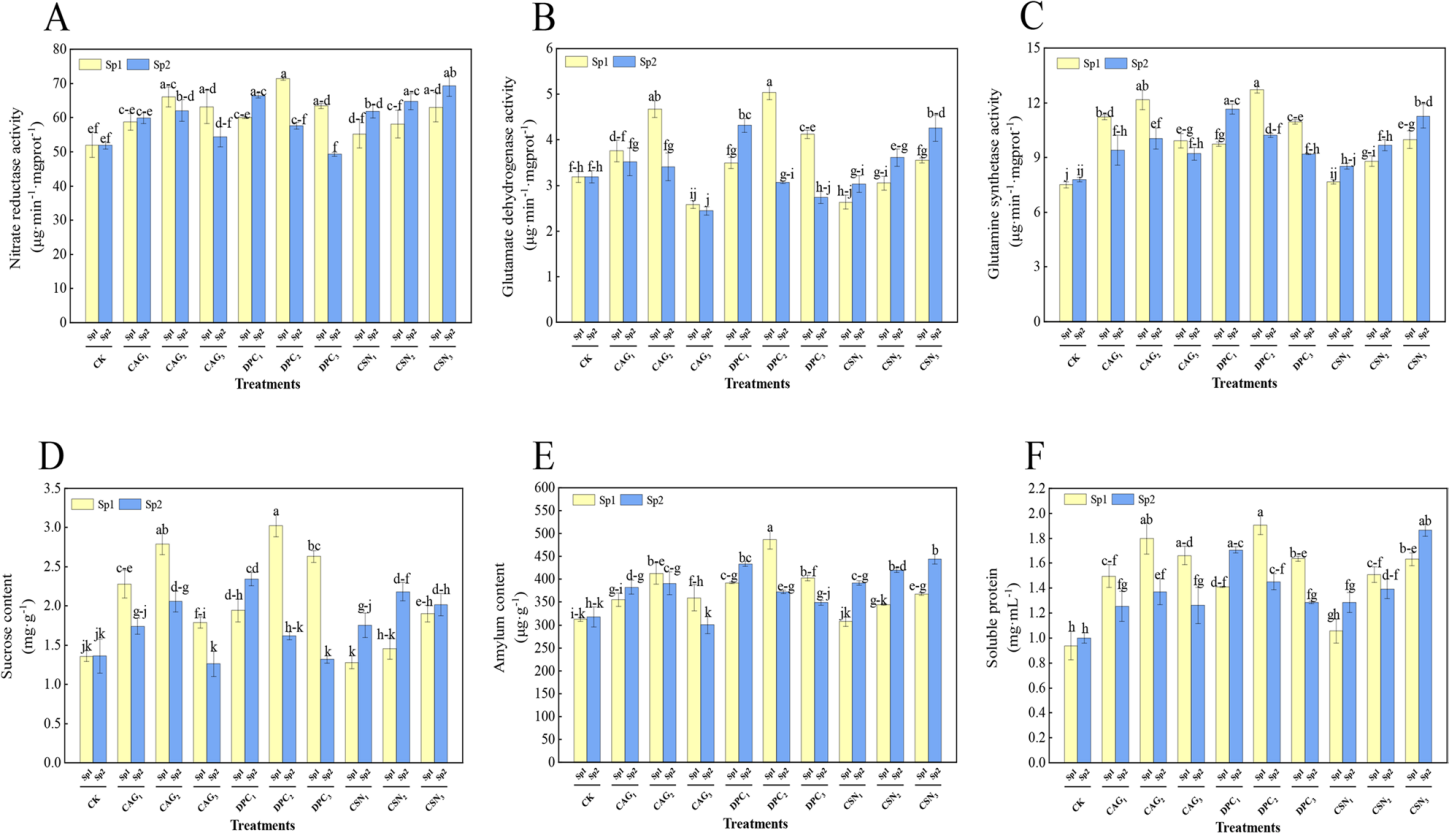
The Effect of PGRs on alfalfa nitrogen Mmtabolism enzymes activity and carbon-nitrogen metabolites. Note: Sp1: primary spray; Sp2: secondary spray; (CK) Water spray; (CAG_1_) 0.5 g L^− 1^ CAG; (CAG_2_) 1.0 g L^− 1^ CAG; (CAG_3_) 1.5 g L^− 1^ CAG; (DPC_1_) 0.25 g L^− 1^ DPC; (DPC_2_) 0.35 g L^− 1^ DPC; (DPC_3_) 0.45 g L^− 1^ DPC; (CSN_1_) 0.15 g L^− 1^ CSN, (CSN_2_) 0.2 g L^− 1^ CSN, (CSN_3_) 0.25 g L^− 1^ CSN. Error bars represent standard deviation (SD); One-way ANOVA was used; Different lowercase letters indicate significant differences between groups (*P* < 0.05)

As shown in Fig. 6, the nitrate reductase activity, glutamate dehydrogenase activity, and glutamine synthetase activity of the DPC treatment showed an increasing and then decreasing trend, and the values were significantly different from those of the CK (*P* < 0.05). The nitrate reductase activity of the DPC_2_ treatment reached 71.43 µg·min^-1^·mgprot^-1^, which was 37.5% higher than that of the CK (Fig. 6A); the glutamate dehydrogenase activity reached 5.04 µg·min^-1^·mgprot^-1^, which was 58.0% higher relative to that of the CK (Fig. 6B); and the glutamine synthetase activity reached the highest value of 12.71 µg·min^-1^·mgprot^-1^, which was increased by 69.4% higher relative to the control (Fig. 6C).

As shown in Fig. 6, the sucrose content and starch content increased successively and then decreased after one spray of DPC, with both showing significant differences (*P* < 0.05) relative to those of the CK. The sucrose content of the DPC_2_treatment reached 3.02 mg·g^-1^, which represented a 52.32% increase relative to that of the CK (Fig. 6D), and the starch content reached 445.46 µg·g^-1^, which represented a 25.24% increase relative to that of the CK (Fig. 6E). Moreover, the soluble protein content of a single spray of the DPC_2_treatment reached the highest value of 1.91 mg·mL^-1^(Fig. 6F). These changes in carbon and nitrogen metabolites may be attributed to the regulatory effect of DPC on key enzymes involved in metabolic pathways. Specifically, DPC could enhance the activity of sucrose phosphate synthase (SPS), a rate-limiting enzyme in sucrose synthesis, thereby promoting sucrose accumulation. For nitrogen metabolism, DPC might upregulate nitrate reductase activity, which is critical for nitrogen assimilation, leading to increased soluble protein content. However, the detailed molecular mechanisms underlying how PGRs modulate these enzyme activities remain to be further elucidated.

### Effect of plant growth regulators treatments on the alfalfa plant strain and seed yield components

**Fig. 7:**
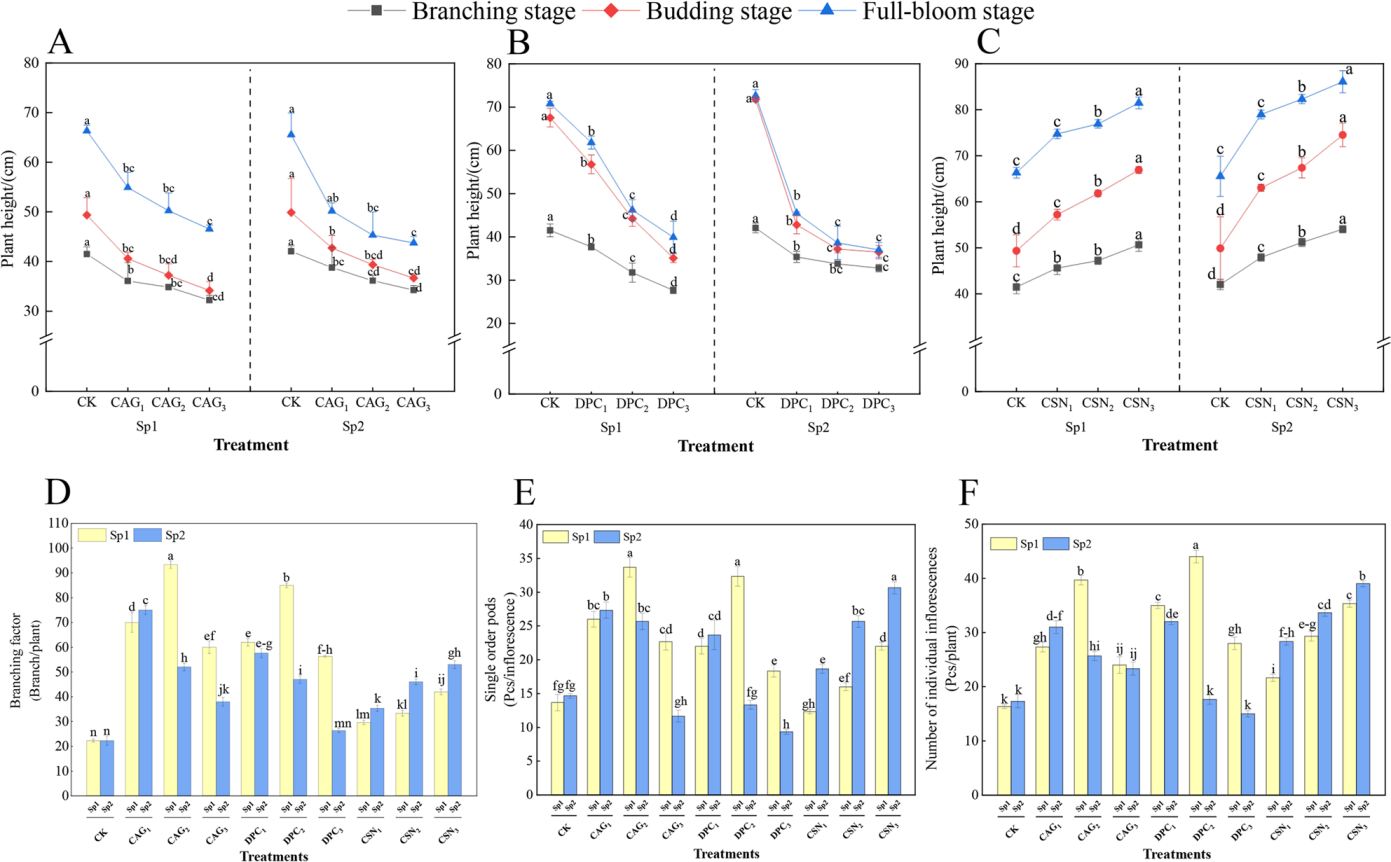
Effect of spraying PGRs on plant height of alfalfa and seed yield componentsNote: Figure 7A, Figure 7B, and Figure 7C show the plant height changes of the CK group vs. the CAG treatment group, the DPC treatment group, and the CSN treatment group, respectively. Sp1: primary spray; Sp2: secondary spray; (CK) Water spray; (CAG_1_) 0.5 g L^-1^ CAG; (CAG_2_) 1.0 g L^-1^ CAG; (CAG_3_) 1.5 g L^-1^ CAG; (DPC_1_) 0.25 g L^-1^ DPC; (DPC_2_) 0.35 g L^-1^ DPC; (DPC_3_) 0.45 g L^-1^ DPC; (CSN_1_) 0.15 g L^-1^ CSN, (CSN_2_) 0.2 g L^-1^ CSN, (CSN_3_) 0.25 g L^-1^ CSN. Error bars represent standard deviation (SD); One-way ANOVA was used; Different lowercase letters indicate significant differences between groups (P<0.05).

As shown in Fig. 7A-C and Supplementary Table S2, after the second spraying of DPC, the plant height of alfalfa treated with DPC3 was the lowest at the branching, bud, and full bloom stages, which were 27.5 cm, 34.13 cm, and 37.03 cm, respectively. Moreover, the findings for each of the periods differed significantly from those of the CK (*P* < 0.05). From the branching to full bloom stage, the plant heights of the CK, DPC1, DPC2, and DPC3 treatments were 25.62 cm, 10.10 cm, 4.83 cm, and 4.23 cm, respectively. Moreover, the plant height decreased as the DPC concentration increased and was lower than that of the CK in all three growth periods after DPC treatment. This indicates that DPC was effective in inhibiting plant height. However, compared with the first spraying, the second spraying had a stronger inhibitory effect on growth and, thus, the latter was more conducive to plant shaping than the former.

As shown in Fig. 7D, the number of branches after one spray of the CAG treatment showed a trend of increasing and then successively decreasing, with the CAG_2_ treatment having the highest number of branches at 93.33 branches/plant. This value was extremely significantly different (*P* < 0.001) from that of the CK. As shown in Fig. 7E-F, the Nps and Nip of the CAG and DPC treatments sprayed one time showed an elevated successive decreasing trend, with the values of the CAG and DPC treatment being significantly different (*P* < 0.05) from that of the CK. Specifically, the CAG_2_ treatment reached 33.67 pods per inflorescence (Fig. 7E) while the DPC_2_ treatment reached 44 inflorescences per plant (Fig. 7F).

### Effect of plant growth regulators treatments on alfalfa seed yield

**Fig. 8:**
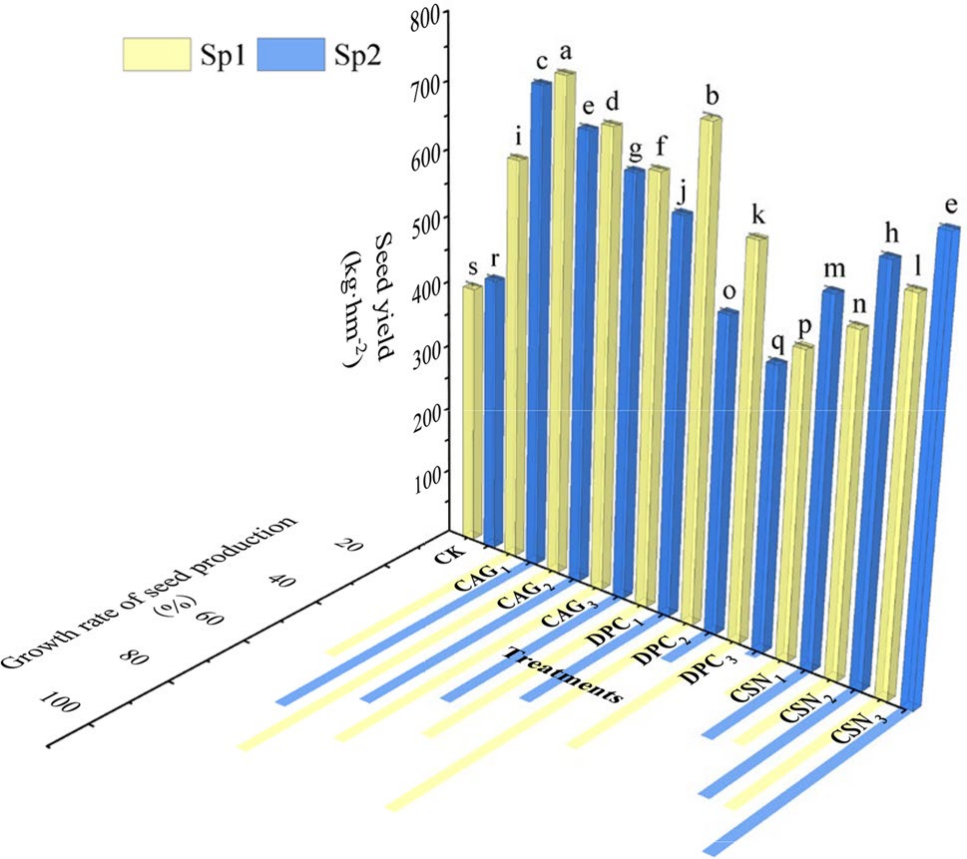
Effect of spraying PGRs on seed yield of alfalfa. Note: Sp1: primary spray; Sp2: secondary spray; (CK) Water spray; (CAG_1_) 0.5 g L^− 1^ CAG; (CAG_2_) 1.0 g L^− 1^ CAG; (CAG_3_) 1.5 g L^− 1^ CAG; (DPC_1_) 0.25 g L^− 1^ DPC; (DPC_2_) 0.35 g L^− 1^ DPC; (DPC_3_) 0.45 g L^− 1^ DPC; (CSN_1_) 0.15 g L^− 1^ CSN, (CSN_2_) 0.2 g L^− 1^ CSN, (CSN_3_) 0.25 g L^− 1^ CSN. Error bars represent standard deviation (SD); One-way ANOVA was used; Different lowercase letters indicate significant differences between groups (*P* < 0.05)

As shown in Fig. 8, the highest seed yield of 751.38 kg·hm^-2^ was obtained after the first spraying of CAG_2_, which represented an increase of 88.4% relative to the CK. A seed yield of 741.21 kg·hm^-2^ was obtained after the first spraying of DPC_2_, which represented an 85.9% increase relative to the CK. The seed yield of the secondary spray of the CSN_3_ treatment was 681.77 kg·hm^-2^, which represented a 61.5% increase relative to the CK. Significant differences in seed yield (*P* < 0.05) were observed between all three treatments and the CK.

### Comprehensive analysis of physiological indicators, plant morphology, and seed yield composition factors in alfalfa after plant growth regulators treatments

#### Comprehensive evaluation of alfalfa physiological indicators, plant morphology, and seed yield composition after treatment with PGRs

The five photosynthetic indicators, seven carbon and nitrogen metabolizing enzymes, three carbon and nitrogen metabolite indicators, and seven seed yield components of alfalfa in the 20 treatments were comprehensively evaluated using the affiliation function. As shown in Supplementary Figure S1, the average affiliation value of the three different DPC after a single spray was higher than that of the CK. Moreover, the average affiliation value of the DPC treatment after a single spray of 0.35 g L^-1^ was 20.29, which was the highest among all treatments.

#### Correlation analysis of physiological indicators, plant morphology, and seed yield composition factors of alfalfa

**Fig. 9:**
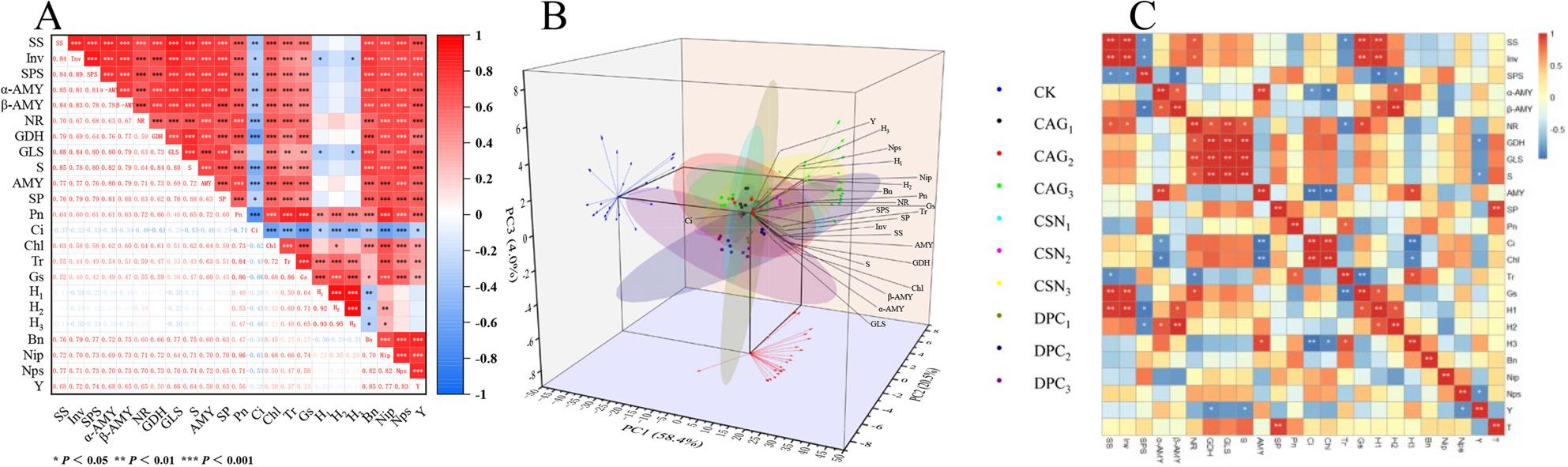
Heatmap of Correlation Between PGRs and Physiological Indicators, Plant Morphology, Seed Yield Components in CARFA and Principal Component Analysis (PCA). Note: SS: Sucrose Synthase; Inv: Invertase; SPS: Sucrose Phosphate Synthase; α-AMY: α-amylase; β-AMY: β-amylase; NR: Nitrate Reductase; GDH: Glutamate Dehydrogenase; GLS: Glutamine Synthetase; S: Sucrose; AMY: Amylase; SP: Soluble Protein; Pn: Net Photosynthetic Rate; Ci: Intercellular CO_2_ Concentration; Chl: Chlorophyll; Tr: Transpiration Rate; Gs: Stomatal Conductance; H_1_: Plant height at branching stage; H_2_: Plant height at bud stage; H_3_: Plant height at full bloom stage; Bn: Branch Number; Nip: Number of Inflorescences per Plant; Nps: Number of Pods per Inflorescence; Y: Seed Yield

According to the correlation analysis results in Fig. 9A, a certain degree of correlation was observed among various indicators. Sucrose synthase (SS), invertase (Inv), sucrose phosphate synthase (SPS), α-amylase (α-AMY), β-amylase (β-AMY), nitrate reductase (NR), glutamate dehydrogenase (GDH), glutamine synthetase (GLS), sucrose (S), amylum (AMY), soluble protein (SP), Pn, chlorophyll (Chl), Tr, Gs, branching factor (Bn), number of pods/inflorescence (Nps), number of inflorescences/plant (Nip), and seed yield (Y) were negatively correlated with the Ci. The branch stage plant height is H_1_, the present bud stage plant height is H_2_, and the flowering stage plant height is H_3_. SS, Inv, SPS, α-AMY, β-AMY, NR, GDH, GLS, S, AMY, SP, Pn, Chl, Tr, and Gs were positively correlated with Bn, Nip, Nps, and Y.

#### Principal component analysis of physiological indicators, plant strains, and seed yield composition factors of alfalfa after plant growth regulators treatments

The results of the principal component analysis (PC1-3; Table S3, Fig. 9B) revealed that PC1 (58.4%), PC2 (20.5%), and PC3 (4.0%) explained 82.9% of the total variance. In PC1, Ci (intercellular CO₂ concentration) was negatively correlated with PC1, while the remaining variables (including photosynthetic rate, plant growth traits, and yield components) were positively correlated. This indicates PC1 reflects a contrast between CO₂ utilization efficiency and overall crop performance; higher PC1 scores suggest stronger photosynthetic capacity, better growth, and improved yield potential. In PC2, NR (nitrate reductase), AMY (amylase), Pn (net photosynthetic rate), Chl (chlorophyll content), Tr (transpiration rate), Gs (stomatal conductance), H_1_(plant height at branching stage), H_2_(bud stage), H_3_(full bloom stage), Nip (number of inflorescences per plant), and Nps (number of pods per inflorescence) were negatively correlated with PC2, and the remaining variables were positively correlated. PC2 thus distinguishes strains with robust nutrient metabolism (nitrogen uptake, carbohydrate breakdown) and photosynthetic activity from those with other advantageous traits, helping identify varieties adapted to specific soil nutrient conditions. In PC3, SPS (sucrose phosphate synthase), NR, Pn, Ci, H_1_, H_2_, H_3_, Bn (branch number), Nip, Nps, and Y (seed yield) were positively correlated with PC3, and the remaining variables were negatively correlated. This component links carbon metabolism, nitrogen utilization, plant growth, and yield traits directly for farmers, PC3 is key to selecting varieties or applying plant growth regulators that enhance traits associated with higher seed yield. Practically, these results guide farmers to prioritize alfalfa varieties with high scores in PC1 (overall performance) and PC3 (yield potential), while using PC2 to choose strains suited to their field’s nutrient availability, ultimately optimizing crop productivity through targeted management.

#### Strain structure equation modeling based on comprehensive analysis

Based Based on comprehensive analysis (correlation, average membership function, PCR principal component analysis), a single spray of 0.35 g L^-1^ showed the best effect. To clarify relationships between photosynthetic characteristics, carbon-nitrogen metabolism, morphological establishment traits and their direct/indirect effects on yield, we constructed a Structural Equation Model (SEM) using representative indicators. Piecewise SEM was applied for path analysis, with the model simplified to adapt to sample size limitations.

**Fig. 10:**
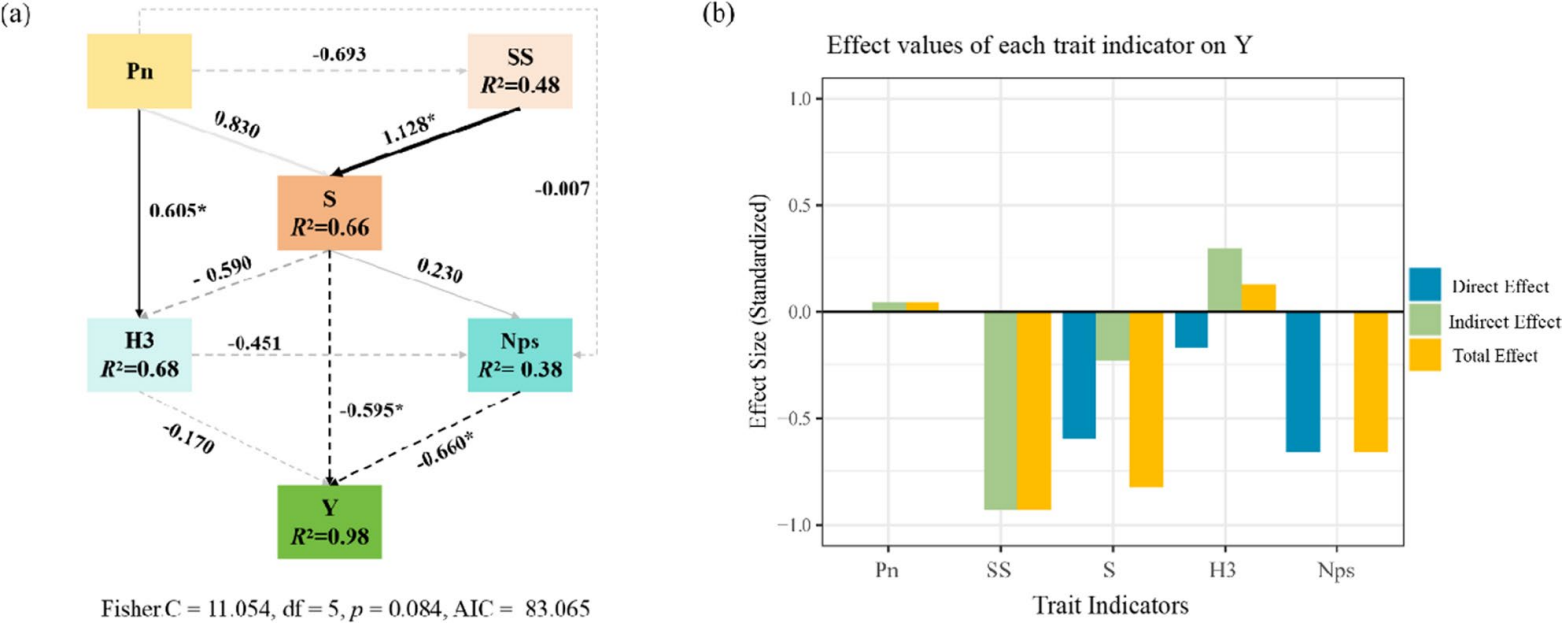
Constructing structural equations for the main parameter measured by DPC on alfalfa. Note: SS: Sucrose Synthase; S: Sucrose; Pn: Net Photosynthetic Rate; H_3_: Plant height at full bloom stage; Nps: Number of Pods per Inflorescence

In model paths (Figs. 9C and 10): Net photosynthetic rate (Pn) had non-significant negative effect on sucrose synthase (SS); strong positive effect on sucrose (S) and plant height at full bloom (H_3_), with H_3_path significant (Estimate = 0.6050, *p* = 0.0458). Photosynthetic efficiency may promote yield via regulating H_3_ farmers can optimize photosynthetic conditions for ideal H_3_. SS had significant positive effect on S (Estimate = 1.1281, *p* = 0.0437), but S had significant negative direct effect on yield (Estimate=-0.5949, *p* = 0.0496) (excess accumulation inhibits yield). Number of pods per inflorescence (Nps) had significant negative effect on yield (Estimate=-0.6596, *p* = 0.0384) farmers should balance pod number to avoid nutrient dilution.

Further analysis of indirect effects: Sucrose synthase (SS) had an indirect effect of -0.927 on yield (transmitted via sucrose (S) and plant height at full bloom (H_3_), with a significantly negative total effect (-0.927) farmers should prioritize varieties with moderate SS activity. Net photosynthetic rate (Pn) had a total effect of 0.042 on yield (non-significant direct path but obvious positive indirect effect) improving photosynthetic efficiency (e.g., proper irrigation or CO₂ supplementation) can indirectly increase yield via enzyme function and growth traits.

Summary: The model revealed the multi-path yield formation mechanism via 0.35 g L^-1^ DPC spray, emphasizing carbon-nitrogen metabolism and morphological establishment as bridges. Guidance for farmers: Select varieties with optimal Pn, balanced H_3_, and moderate Nps; adjust field management to regulate sucrose levels and enzyme activity; prioritize traits with positive indirect effects on yield. These strategies optimize alfalfa productivity through targeted interventions.

## Discussion

### Effect of sprayed PGRs on ultrastructure of alfalfa leaves

Plant cells are involved in the absorption, transformation, and transmission of light energy during photosynthesis [20, 21], and enhanced photosynthetic capacity can promote the synthesis and accumulation of organic matter to a certain extent [22]. This experiment analyzed electron microscope images of alfalfa leaves sprayed with different types of plant growth regulators (PGRs). The results showed that PGR treatments increased the number of starch grains in window cells, reduced the grana and stroma lamellae in chloroplasts, and decreased the number of cristae in mitochondria. In addition, the increase in PGR dosage and differences in types enhanced the impact on leaf ultrastructure, leading to the expansion of chloroplast lamellae and the differentiation or disappearance of mitochondrial cristae.The increase in starch grain number is not just a passive byproduct of enhanced photosynthesis; it has important implications for alfalfa cultivation: as the main carbon reserve, accumulated starch supports later reproductive growth stages (such as flowering and pod development), directly aligning with the goal of increasing seed yield in alfalfa production systems. Alfalfa may be more sensitive to excessive PGR exposure, emphasizing the need for precise control of concentration in actual cultivation to balance photosynthetic gains and cell structural stability.

The above findings indicate that with the increase in the number of applications and concentration of PGRs, the treatment affects the normal growth and development of leaves and damages chloroplasts and mitochondria in leaves to a certain extent. Integrating these results into a broader framework of PGR use, we believe that moderate application of PGRs can enhance carbon storage through starch grains to increase yield, while excessive use may damage the photosynthetic machinery these insights refine current recommendations for sustainable alfalfa management.

### Effect of spraying PGRs on photosynthetic capacity of alfalfa

Photosynthesis is the process by which crops produce energy and exchange substances with the external environment, and its impact on plant growth, development, and yield is one of the most fundamental activities of this process [23]. Plant growth regulators (PGRs) can improve the photosynthetic utilization efficiency of crops and facilitate dry matter accumulation which is crucial for increasing forage yield and quality in alfalfa cultivation, as biomass production is the core goal. Therefore, organic nutrients in grains are effectively accumulated, leading to an increase in yield [24, 25].

In this study, cell ultrastructure images showed that there were differences in the degree of damage to leaf cells and their organelle structures after spraying different types of PGRs. After spraying 1.0 g·L⁻¹ CAG and 0.35 g·L⁻¹ DPC once, followed by spraying 0.25 g·L⁻¹ CSN once, the damage to leaf cells and their organelle structures was minor, and the plants could perform normal photosynthesis. This combined treatment caused the least damage to cells while improving photosynthetic parameters (net photosynthetic rate Pn, chlorophyll content Chl, transpiration rate Tr, stomatal conductance Gs), filling the gap in the current alfalfa PGR application framework: although single PGR application is common, our results emphasize that the sequential combination of CAG, DPC, and CSN can strike a balance between promoting growth and maintaining structural integrity which is crucial for sustainable cultivation, as long-term leaf health supports multiple harvests. CAG and DPC can increase plant compactness and improve ventilation and light transmission [13, 15], thereby reducing interplant shading in dense alfalfa populations (a common challenge in high-yield cultivation). In addition, CSN can enhance cell activity and improve leaf photosynthetic capacity [18], thereby increasing Pn and Chl contents. Spraying 1.0 g·L⁻¹ CAG and 0.35 g·L⁻¹ DPC once, followed by 0.25 g·L⁻¹ CSN once, can increase leaf Tr, raise leaf Gs, and reduce intercellular CO₂ concentration Ci and other indicators.

Key comparisons with existing literature Our study found: Wang Yuan [24] showed that the net photosynthetic rate (Pn), stomatal conductance (Gs), and transpiration rate (Tr) after one application of CAG were better than those after two applications, which is consistent with our findings excessive PGR application (such as multiple single treatments) may not be the optimal choice. However, our combined treatment, by integrating the cell activation effect of CSN, was superior to Wang’s single application, indicating that sequential use of complementary PGRs is more effective than repeated use of the same compound. Yang et al. [25] also found that flusilazole treatment increased leaf chlorophyll content and maintained it at a high level, prolonged photosynthesis time, and improved population photosynthetic capacity which is similar to our CSN-induced chlorophyll retention, but our study extended this to alfalfa (a forage crop with a different growth cycle from the grains studied by Yang). Liu et al. [26] showed that DPC induces chlorophyll synthesis, increases chlorophyll content, and improves light absorption which is consistent with our DPC results, but our combination added the structural benefits of CAG, making it more suitable for dense alfalfa canopies. He et al. [27] also found that after 1,1-DPC treatment, the photosynthetic capacity and chlorophyll content of plants were significantly increased; while Tang et al. [28] found that sodium nitrophenolate promoted the growth and development of Chlorella vulgaris our study bridges these findings in aquatic and terrestrial plants, proving that PGR-induced photosynthetic enhancement has a conserved nature, but needs to be optimized for crops (such as alfalfa’s need for tolerance to multiple harvests).

### Effect of spraying PGRs on carbon and nitrogen metabolism capacity in alfalfa leaves

During the growth and maturation of plants, the intensity, coordination, and dynamic change trends of carbon and nitrogen metabolism indirectly or directly affect the content and composition ratio of various chemical components. These changes have significant impacts on plants for the perennial forage alfalfa, both yield (dry matter accumulation) and quality (proteins from nitrogen metabolism, energy from carbon compounds) are directly related to metabolic efficiency, which is the core of its commercial value. In addition, the balance and coordination of carbon and nitrogen metabolism are key factors in the transition from vegetative growth to reproductive growth in plants [29–31]. Metabolites such as sucrose, starch, and soluble proteins can serve as markers for carbon assimilation [32–34], nitrogen transformation [35]– [36], and material synthesis, transport, and accumulation in leguminous crops [37, 38].

In soybeans, Zheng et al. [39] found that the use of plant growth regulators can regulate physiological indicators related to nitrogen metabolism in leaves, significantly increasing yield components such as the number of grains per plant and grain weight. Luo et al. [40] showed that foliar spraying of plant growth regulators during the full flowering stage of soybeans can increase the activities of sucrose phosphate synthase, sucrose synthase, and invertase in leaves, coordinate the dynamic balance of carbon and nitrogen metabolism in various organs, promote pod setting and reduce pod drop, thereby significantly increasing the number of effective pods per plant and yield of soybeans. While these studies highlight the metabolic benefits of plant growth regulators in annual leguminous crops, our findings for the perennial forage alfalfa add key context: unlike soybeans, which are harvested once a year, alfalfa needs to regenerate multiple times after cutting thus, protecting leaf cell structure and long-term metabolic capacity is as important as short-term yield gains. Liu et al. [41, 42] found that foliar spraying of DTA-6 and S3307 can delay leaf senescence, promote leaf cell activity in the late reproductive stage of soybeans, increase the intensity of carbon and nitrogen metabolism in pods, and drive the transfer of sucrose, fructose, and starch from leaves to pods, thereby allocating more carbohydrates to pod formation and increasing soybean yield. For alfalfa, the transfer of metabolites from leaves to storage organs (roots) is crucial for regeneration, so the optimal plant growth regulator dose must balance current metabolic gains with future regeneration potential.

**Fig. 11:**
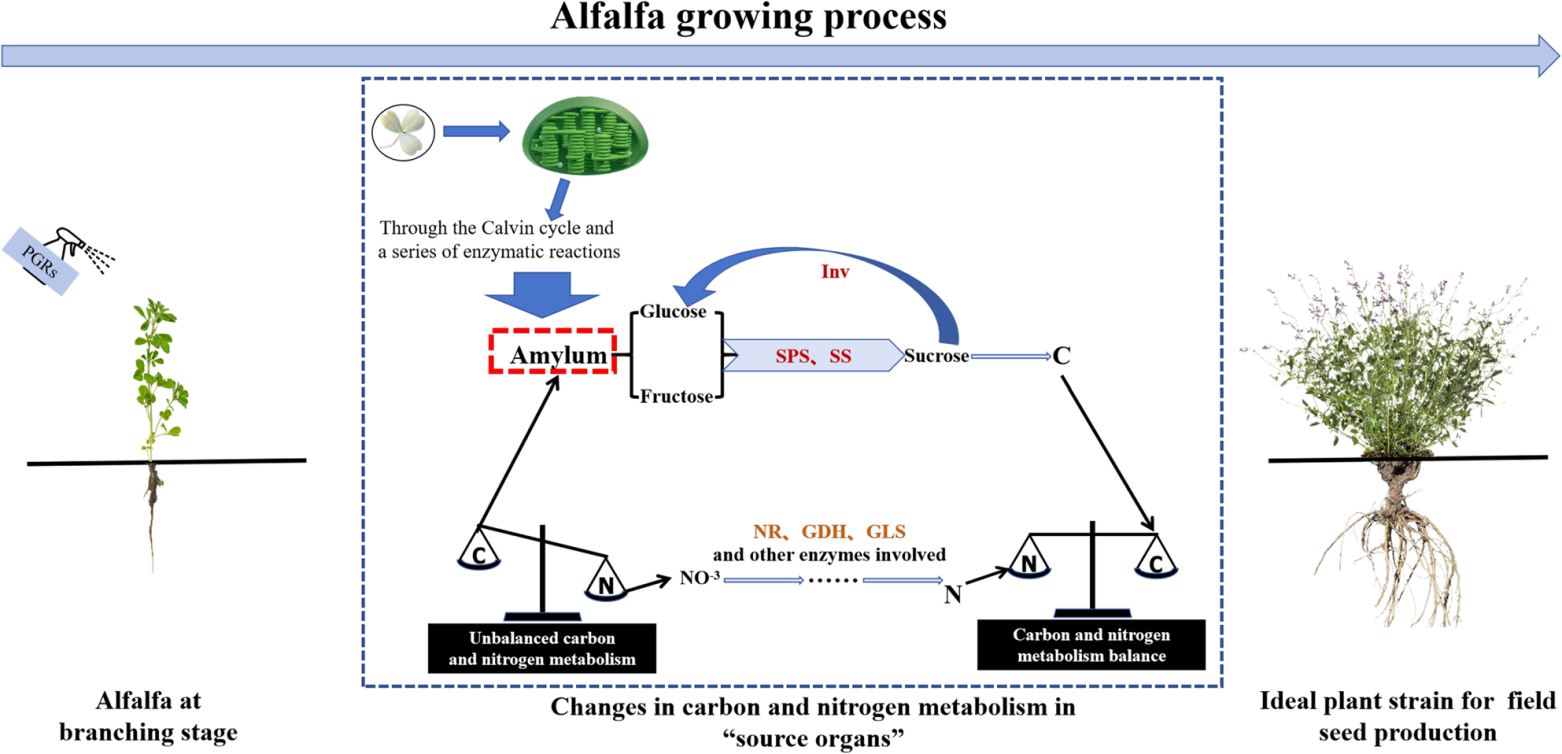
Effect of carbon and nitrogen balance on the growth of alfalfa

This study shows that spraying three different types of plant growth regulators (PGRs) can improve the carbon and nitrogen metabolism capacity of alfalfa. Among them, when CAG and DPC are sprayed once at concentrations of 1.0 g·L⁻¹ and 0.35 g·L⁻¹ respectively, the activities of carbon and nitrogen metabolic enzymes and their product contents are relatively high. These single doses meet the actual management needs of alfalfa applying once before the first cutting can promote metabolism without damaging cells, while supporting immediate forage yield and post-harvest regeneration. However, with the increase in the number of applications and concentration of PGRs, the carbon and nitrogen metabolism capacity decreases significantly, which is attributed to the destruction of plant leaf cell structure. This situation will hinder the conversion of light energy into chemical energy by plant source organs (source-sink theory) [43] for alfalfa, this destruction means that leaves cannot allocate carbohydrates to roots for regeneration, weakening the crop’s persistence after multiple harvests. In addition, with organelle damage, photosynthetic capacity decreases, and normal carbon and nitrogen metabolism cannot be carried out. Therefore, the activity of carbon and nitrogen metabolic enzymes decreases, and related metabolites also decrease, leading to an imbalance in carbon and nitrogen metabolism (Fig. 11).For CSN treatment, carbon and nitrogen metabolism increases with the number of treatments and concentration, reaching the highest enzyme activity and metabolite content after the second spraying of 0.25 g·L⁻¹. This is because CSN is a potent cell activator that can significantly enhance cell activity. Therefore, the 0.25 g·L⁻¹ CSN treatment twice shows an upward trend, although the amount of carbon and nitrogen metabolites does not decrease to the same extent with the increase in the number of treatments and concentration. This unique trend makes CSN very promising in alfalfa applications: its ability to maintain metabolic gains during repeated applications indicates that it can be used to accelerate leaf regeneration after harvest, which is a gap in current PGR recommendations for forage crops. Subsequent studies should clarify the trends of CSN after further treatments and concentration increases to optimize its application in the continuous production cycle of alfalfa.

In summary, spraying PGRs can improve the carbon and nitrogen metabolism of plants, but the number of applications and optimal concentration need to be determined this is crucial for balancing the immediate yield, quality, and long-term sustainability of alfalfa.

### Effect of applying PGRs on alfalfa plant strain

In this study, the concentration and frequency of spraying PGRs affected the constitutive factors for seed yield and plant growth. Moreover, the concentration of CAG and DPC inhibited or promoted alfalfa growth. A second spray of 1.5 g L^− 1^ CAG and 0.45 g L^− 1^ DPC effectively inhibited the growth of alfalfa, and the number of branches of these two treatments decreased by 59.3% and 69.1% compared with that of the first spray of 1.0 g L^− 1^ of CAG and 0.45 g L^− 1^ of DPC, respectively. These results are not optimal for the construction of the plant strain. The ideal field seed production plant strain is shown in Supplementary Figure S2. Thus, the plant must be dwarfed while ensuring that the number of branches increases. However, one spray of CAG_2_ led to the highest number of branches among all treatments and decreased plant height by 32.8% compared with that of the CK. Moreover, one spray of the DPC_2_ treatment increased the number of branches, with this treatment ranking second among all treatments. However, plant height decreased by 34.7% compared with that of the CK. Zhao et al. [44] found that different amounts of flumetralin treatment inhibited the plant height of island cotton to different degrees, which could improve the balance between vegetative growth and reproductive growth. Cai et al. [45] demonstrated that the application of flumetralin to cotton inhibited the growth of the terminal buds of the main stems and leafy shoots, increased the height of the plant and the number of fruiting shoots, and reduced the leaf branch length and average internode length, which effectively increased cotton yield, with 25% flumetralin at 300 ml·hm^− 2^ leading to high yields in seed cotton. Wang et al. [14] showed that chemical topping with flutriafol effectively controlled the height of cotton plants, shaped a good plant strain, and improved cotton yield. Zhang et al. [46] found that 25% flutriafol suspension can replace manual topping and led to better growth and development of cotton in the concentration range of 1,050 − 1,200 ml·hm^− 2^; thus, this treatment had a good regulatory effect on and promoted seed cotton yield. Zhang et al. [47] found that the scientific and rational use of acetamiprid improved the germination rate and seedling vigor of cotton, improved the shape of cotton, enhanced photosynthesis, and reduced pests and diseases during growth, thereby improving cotton growth, yield, and quality. Gao et al. [48] demonstrated that spraying 240 mg L^− 1^ acetamiprid led to the highest seed yield and had the best effect on increasing yield. He et al. [27] showed that the photosynthetic capacity and chlorophyll content of plants were significantly improved after treatment with acetamiprid, with the height of the plants significantly reduced, the number of pods per plant increased, seed size increased, and the yield improved.

### Effect of spraying PGRs on alfalfa plant strain and its constituent factors

In this study, CAG and DPC treatments inhibited the growth and development of alfalfa to varying degrees which is crucial for seed production, as moderate inhibition can reduce lodging (a major obstacle to high seed yield) and redirect photosynthetic resources from excessive vegetative growth to seed filling. The second spray of DPC₃ had the best inhibitory effect on plant growth, resulting in the lowest growth rate, but this excessive inhibition ultimately reduced seed yield (consistent with earlier findings on cell damage), highlighting the need for balanced PGR application. Spraying CAG once at 1.0 g L^-1^ and DPC once at 0.35 g L^-1^ increased the contents of amylase, sucrose, andsolubleprotein due to enhanced photosynthetic capacity. These changes are biologically significant: starchsupports seed germination, sucroseprovides energy for early seedling growth, andsoluble proteinenhances seed vigor these traits not only increase yield but also improve the market value of seeds. Promoting vegetative growth in the early stage provides a material basis for later growth, leading to the highest seed yields of 751.38 kg·hm⁻² and 741.21 kg·hm⁻² respectively making these treatments economically feasible for seed producers. However, a single spray of CSN promoted the plant height of alfalfa, and the second spray of CSN₃ resulted in Nip and Nps being much higher than the control (CK). These changes increased seed yield, indicating that CSN improved photosynthetic capacity, promoted cell activity, increased the efficiency of converting light energy into chemical energy, adjusted the carbon-nitrogen metabolism balance, balanced vegetative and reproductive growth, reduced energy loss, improved the utilization rate of carbon-nitrogen metabolites, and promoted the accumulation of carbon-nitrogen metabolites. In addition, under this treatment, the seed yield reached 681.77 kg·hm⁻² still higher than the average level, showing the potential of CSN for those who prefer growth-promoting PGRs rather than inhibitors. Siebert et al. [49] found that PGRs increased cotton seed and lint yields similar to our yield gains, but cotton yield includes fiber, while we focus on alfalfa seed yield, so traits unrelated to cotton such as the number of seeds per pod (Nps) are prioritized. Hu et al. [50] studied eggplant and found that combined PGR treatments increased yield while our second spray of CSN showed that consecutive single treatments are also effective, providing farmers with a simpler and more flexible option without the need to mix chemicals. An Xia et al. [17] found that sodium dinitrophenolate increased wheat yield through dry matter accumulation similar to the effect of CSN, but wheat is an annual grain, so our results must consider the perennial characteristics of alfalfa.

In summary, screening the optimal concentrations of PGRs is necessary to promote the vegetative and physiological growth of different plants for alfalfa seed production, a single application of CAG (1.0 g L^-1^) and DPC (0.35 g L^-1^) balances growth inhibition and resource allocation to achieve high yield and quality, aligning with sustainable agricultural practices by reducing over-application and maximizing input efficiency. These findings fill a gap in alfalfa PGR research, where most studies focus on forage yield rather than seed production, and provide feasible recommendations for farmers to increase seed yield and profitability.

### Construction of equations for the structure of alfalfa strains for seed production

In this study, the net photosynthetic rate had a negative effect on sucrose synthase, although not significant (*p* = 0.127), with an estimated value of -0.69. This finding is meaningful for alfalfa cultivation and PGR use: while PGRs like CAG improved photosynthetic capacity, excessivenet photosynthetic rate(as observed in over-applied DPC treatments) without correspondingsucrose synthaseactivity would lead to sugar accumulation inhibiting growth highlighting the need to balance PGR doses to match photosynthetic output with enzymatic capacity.

The net photosynthetic rate had a significant positive effect on plant height (H_3_) (*p* = 0.046), indicating that higher photosynthetic rates are associated with more vigorous plant growth. For alfalfa seed production, this underscores the value of plant growth regulators (PGRs) like CAG and DPC: they reduce H_3_(to prevent lodging) while maintainingnet photosynthetic rate, balancing growth vitality with lodging resistance a key trade-off in high-yield seed cultivation.

Sucrose synthase had a significant positive effect on sucrose content (*p* = 0.044), confirming that sucrose synthase activity directly drives the formation of carbon assimilation products. This aligns with our PGR results: CAG and DPC treatments increasedsucrose synthaseactivity, leading to higher sucrose levels and seed yields linking enzymatic function to practical yield gains for farmers.

Although the negative effect of sucrose content on plant height (H_3_) (-0.59) was not significant, it suggests that high sugar accumulation may inhibit vegetative growth a sugar signaling phenomenon reported in legumes like soybeans [51–53]. For alfalfa, this result emphasizes the need to use PGRs (such as CSN) that enhance sucrose transport to seeds (sink) rather than just synthesis in leaves (source) to avoid growth inhibition. More importantly, the direct effect of sucrose content on yield was significant (estimated value=-0.5949,*p* = 0.0496) and negative, reflecting source-sink imbalance: vigorous sugar synthesis in leaves (source) but limited transport to seeds (sink). This is a key challenge in alfalfa seed production our excessive PGR treatments had this issue, while CSN treatments alleviated it by increasing sink strength (more pods), highlighting CSN’s unique value in balancing source and sink.

The path effect of the number of pods per inflorescence (Nps) on yield was significant (*p* = 0.038), emphasizing that reproductive organ formation is a direct driver of yield.This study’sCSN treatments increased Nps, consistent with this result, explaining why CSN can improve seed yield even with moderate growth promotion.

Although the direct path of Pn on yield was negative (-0.1701) and not significant, the total effect of Pn on yield was positive (0.0423), indicating that it contributes indirectly to yield through upstream processes (SS→S→Nps/H_3_). This indirect contribution is crucial for PGR strategies: PGRs do not need to directly increase yield they can optimize upstream physiological processes to enhance output, as shown in our CAG and DPC treatments.

This structural equation model (SEM) analysis reinforced the conclusions ofthisstudy by linking physiological mechanisms to practical plant growth regulator (PGR) recommendations for alfalfa seed production, filling the gap in current legume forage literature that lacks such mechanistic insights.

## Conclusion

In summary, this study’s main findings include two key plant growth regulator (PGR) treatments for alfalfa seed yield improvement: a single spray of 1.0 g L^-1^ CAG during the early branching stage achieved the highest branch number (93.33 per plant), number of inflorescences per plant, and seed yield (751.38 kg·hm^-2^); while a single spray of 0.35 g L^-1^ DPC during the branching stage (50% of plants showing initial branching) optimized leaf cellular ultrastructure (intact chloroplasts and mitochondria with neatly arranged cristae), enhanced photosynthetic characteristics (net photosynthetic rate, intercellular CO_2_ concentration, transpiration rate, chlorophyll content, and stomatal conductance all superior to control), and increased carbon/nitrogen metabolic enzyme activities and metabolite contents, resulting in a seed yield of 741.21 kg·hm^-2^. Limitations of this study include the focus on single PGR applications (no combined treatments), lack of long-term assessments on soil health or crop rotation impacts, and limited testing across alfalfa varieties and environmental conditions. Future research should explore combined PGR treatments, multi-year trials to evaluate sustainability, variety-specific responses, and the molecular mechanisms linking PGR-induced changes in ultrastructure/photosynthesis to branching and yield traits.

## Supplementary Information

Supplementary Material 1.

## Data Availability

Data were used for the research described in the article. Data is provided within the manuscript or supplementary information files.
