# Supplementary material for: Modulating alfalfa growth: impacts of plant growth regulators on physiology, architecture, and seed yield
Source: BMC Plant Biol. 2026 Feb 6;26:448. doi: 10.1186/s12870-026-08173-x (PMC12973799; doi:10.1186/s12870-026-08173-x)
Supplement: Supplementary file 1 — Supplementary Material 1. [file 12870_2026_8173_MOESM1_ESM.docx]

**Table S1 Preparation of Treatment Solution Concentrations**

| **PGRs** | **Reagent concentration(g·L^-1^)** | | | |
| --- | --- | --- | --- | --- |
| **CAG** | 0 | 0.50 | 1.00 | 1.50 |
| **DPC** | 0 | 0.25 | 0.35 | 0.45 |
| **CSN** | 0 | 0.15 | 0.20 | 0.25 |

Note: PGRs is plant growth regulators.

The three concentrations of CAG are recorded from low to high as CAG_1_, CAG_2_ and CAG_3_; DPC is the same as above, respectively recorded as DPC_1_, DPC_2_, DPC_3_; CSN is the same as above, respectively, CSN_1_, CSN_2_ and CSN_3_.

**Table S2 Significance of the effect of spraying PGRs on alfalfa plant height**

| **The number of spraying** | **Treatment** | **Growth period** | | | |
| --- | --- | --- | --- | --- | --- |
|  |  | **Branch period** | **Squaring stage** | **Flowering** |  |
| Sp1 | CK | 40.70±0.36f | 47.57±0.54f | 70.8±0.60f |  |
| Sp1 | CAG1 | 36.43±0.98gh | 40.63±0.70hi | 54.9±1.78h |  |
| Sp1 | CAG2 | 34.83±0.32hi | 39.57±1.39ij | 47.57±1.18ij |  |
| Sp1 | CAG3 | 32.23±0.55i | 34.17±1.03m | 46.57±0.45jk |  |
| Sp1 | DPC1 | 38.53±0.88fg | 56.77±1.26d | 61.8±0.87g |  |
| Sp1 | DPC2 | 34.33±1.43hi | 44.23±1.03g | 46.23±1.39jk |  |
| Sp1 | DPC3 | 32.30±1.95i | 36.50±0.91lm | 39.23±1.10l |  |
| Sp1 | CSN1 | 45.63±0.83e | 57.23±0.67d | 72.03±0.91ef |  |
| Sp1 | CSN2 | 47.23±0.47e | 61.83±0.41c | 76.00±0.78cd |  |
| Sp1 | CSN3 | 50.67±0.81d | 66.93±0.42b | 79.23±0.17b |  |
| Sp2 | CK | 47.05±1.00e | 51.80±0.26e | 72.67±0.30ef |  |
| Sp2 | CAG1 | 38.43±0.41fg | 42.77±1.44gh | 50.20±0.95i |  |
| Sp2 | CAG2 | 36.17±0.61gh | 39.37±0.62ijk | 44.00±0.70k |  |
| Sp2 | CAG3 | 34.23±0.55hi | 36.70±0.32klm | 39.23±0.64l |  |
| Sp2 | DPC1 | 36.30±1.01gh | 42.80±1.19gh | 45.50±0.17jk |  |
| Sp2 | DPC2 | 32.53±1.19i | 37.20±0.49jkl | 38.83±1.33l |  |
| Sp2 | DPC3 | 27.50±0.35j | 34.13±0.55m | 37.03±0.99l |  |
| Sp2 | CSN_1_ | 57.23±0.67c | 63.07±0.43c | 74.03±1.36de |  |
| Sp2 | CSN_2_ | 61.83±0.41b | 67.40±1.27b | 78.57±1.13bc |  |
| Sp2 | CSN_3_ | 66.93±0.42a | 74.53±1.49a | 86.10±1.39a |  |
| **Significance** | | *F* *P* | *F* *P* | *F* *P* |  |
| **Sp** | | \| 102.853 \| *** \| \| --- \| --- \| | \| 0.075 \|  \| \| --- \| --- \| | \| 43.54 \| *** \| \| --- \| --- \| |  |
| **Treatment** | | \| 302.109 \| *** \| \| --- \| --- \| | \| 349.599 \| *** \| \| --- \| --- \| | \| 467.147 \| *** \| \| --- \| --- \| |  |
| **Sp*Treatment** | | \| 41.622 \| *** \| \| --- \| --- \| | \| 24.773 \| *** \| \| --- \| --- \| | \| 18.839 \| *** \| \| --- \| --- \| |  |

Note: Sp is the number of sprays; ***: strong significant difference; **: significant difference; *: significant difference.

**Table S3 Eigenvalue and percentage of variance for the first five axes derived from PCA and correlation between the axes and the variables**

| **Variable** | **PC1** | **PC2** | **PC3** | **PC4** | **PC5** |
| --- | --- | --- | --- | --- | --- |
| **Eigenvalue** | 13.42996 | 4.72218 | 0.92499 | 0.87456 | 0.50198 |
| **Variance** | 58.39112 | 20.53124 | 4.02168 | 3.80244 | 2.18254 |
| **SS** | 0.24821 | -0.07982 | -0.09179 | 0.07443 | 0.22680 |
| **Inv** | 0.23882 | -0.15003 | -0.04816 | 0.11798 | -0.11080 |
| **SPS** | 0.23914 | -0.11993 | 0.00709 | 0.16186 | -0.14604 |
| **α-AMY** | 0.23969 | -0.09113 | -0.14375 | 0.10783 | 0.17515 |
| **β-AMY** | 0.23760 | -0.09631 | -0.11385 | 0.04656 | 0.12127 |
| **NR** | 0.22029 | 0.04222 | 0.09437 | 0.12108 | -0.37650 |
| **GDH** | 0.22932 | -0.01673 | -0.19113 | -0.36514 | 0.38309 |
| **GLS** | 0.22673 | -0.17083 | -0.05724 | 0.07684 | 0.26558 |
| **S** | 0.23951 | -0.09331 | -0.11612 | -0.25872 | 0.10012 |
| **AMY** | 0.23538 | 0.00551 | -0.16520 | 0.08241 | 0.05742 |
| **SP** | 0.22195 | -0.06359 | -0.05981 | 0.30131 | 0.01390 |
| **Pn** | 0.22687 | 0.21451 | 0.01124 | -0.02492 | -0.15113 |
| **Ci** | -0.15583 | -0.20805 | 0.05067 | 0.66054 | 0.25228 |
| **Chl** | 0.20526 | 0.12309 | -0.36526 | -0.04901 | -0.47187 |
| **Tr** | 0.18484 | 0.25188 | -0.20852 | 0.30008 | -0.13295 |
| **Gs** | 0.18656 | 0.30666 | -0.08176 | 0.01933 | 0.18323 |
| **H1** | 0.00327 | 0.43210 | 0.13273 | 0.17057 | 0.23948 |
| **H2** | 0.03465 | 0.44235 | 0.03670 | 0.09461 | 0.06511 |
| **H3** | 0.00986 | 0.44410 | 0.16815 | -0.02222 | 0.11217 |
| **Bn** | 0.21793 | -0.18322 | 0.32672 | -0.15956 | -0.08374 |
| **Nip** | 0.24370 | 0.10388 | 0.24424 | -0.03118 | -0.03021 |
| **Nps** | 0.23731 | 0.00444 | 0.39230 | -0.12869 | 0.17901 |
| **Y** | 0.21061 | -0.09590 | 0.55561 | 0.10414 | -0.14994 |


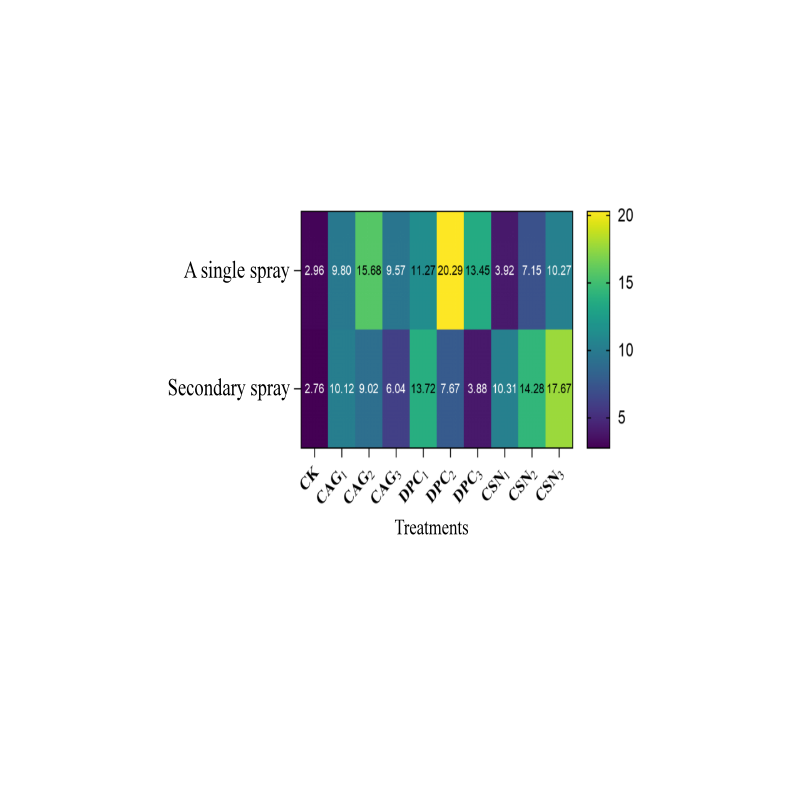


### **Figure** S1. **Heat evaluation of PGRs on physiological indicators, plant morphology, and seed yield composition factors in alfalfa**


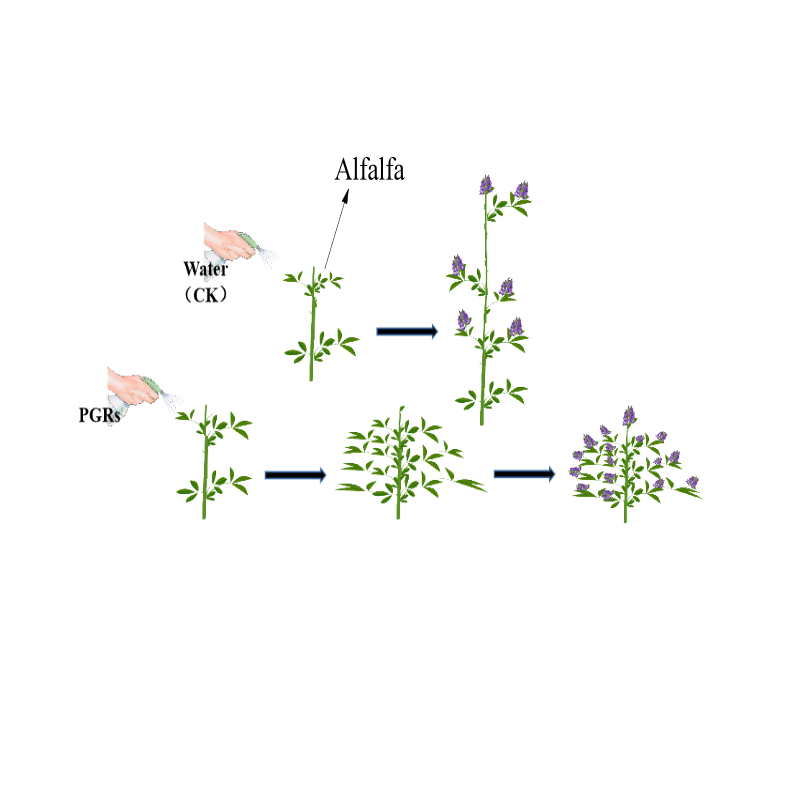


**Figure S2**. **Ideal plant strain for field seed production**
